# Recent Trends and Applications of Nanoencapsulated Bacteriocins against Microbes in Food Quality and Safety

**DOI:** 10.3390/microorganisms11010085

**Published:** 2022-12-28

**Authors:** Bakhtawar Shafique, Muhammad Modassar Ali Nawaz Ranjha, Mian Anjum Murtaza, Noman Walayat, Asad Nawaz, Waseem Khalid, Shahid Mahmood, Muhammad Nadeem, Muhammad Faisal Manzoor, Kashif Ameer, Rana Muhammad Aadil, Salam A. Ibrahim

**Affiliations:** 1 Institute of Food Science and Nutrition, University of Sargodha, Sargodha 40100, Pakistan; 2 College of Food Science and Technology, Zhejiang University of Technology, Hangzhou 310014, China; 3 Shenzhen Key Laboratory of Marine Microbiome Engineering, Institute for Advanced Study, Shenzhen University, Shenzhen 518060, China; 4 Institute for Innovative Development of Food Industry, Shenzhen University, Shenzhen 518060, China; 5 Department of Food Science, Government College University Faisalabad, Faisalabad 38000, Pakistan; 6 Guangdong Provincial Key Laboratory of Intelligent Food Manufacturing, Foshan University, Foshan 528011, China; 7 National Institute of Food Science and Technology, University of Agriculture, Faisalabad 38000, Pakistan; 8 Food Microbiology and Biotechnology Laboratory, North Carolina Agricultural and Technical State University, Greensboro, NC 27411, USA

**Keywords:** bacteriocins, lactic acid bacteria, Gram-negative bacteria, Gram-positive bacteria, nanoencapsulation, antimicrobial activity

## Abstract

Bacteriocins are ribosomal-synthesized peptides or proteins produced by bacterial strains and can inhibit pathogenic bacteria. Numerous factors influence the potential activity of bacteriocins in food matrices. For example, food additives usage, chemical composition, physical conditions of food, and sensitivity of proteolytic enzymes can constrain the application of bacteriocins as beneficial food preservatives. However, novel bacteriocin nanoencapsulation has appeared as an encouraging solution. In this review, we highlight the bacteriocins produced by Gram-negative bacteria and Gram-positive bacteria including lactic acid bacteria that have shown positive results as potential food preservatives. In addition, this review encompasses the major focus on bacteriocins encapsulation with nanotechnology to enhance the antimicrobial action of bacteriocins. Several strategies can be employed to encapsulate bacteriocins; however, the nanotechnological approach is one of the most effective strategies for avoiding limitations. Nanoparticles such as liposomes, chitosan, protein, and polysaccharides have been discussed to show their importance in the nanoencapsulation method. The nanoparticles are combined with bacteriocins to develop the nano-encapsulated bacteriocins from Gram-negative and Gram-positive bacteria including LAB. In food systems, nanoencapsulation enhances the stability and antimicrobial functionality of active peptides. This nanotechnological application provides a formulation of a broad range of antimicrobial peptides at the industry-scale level. Nano-formulated bacteriocins have been discussed along with examples to show a broader antimicrobial spectrum, increase bacteriocins’ applicability, extend antimicrobial spectrum and enhance stability.

## 1. Introduction

**Figure 1:**
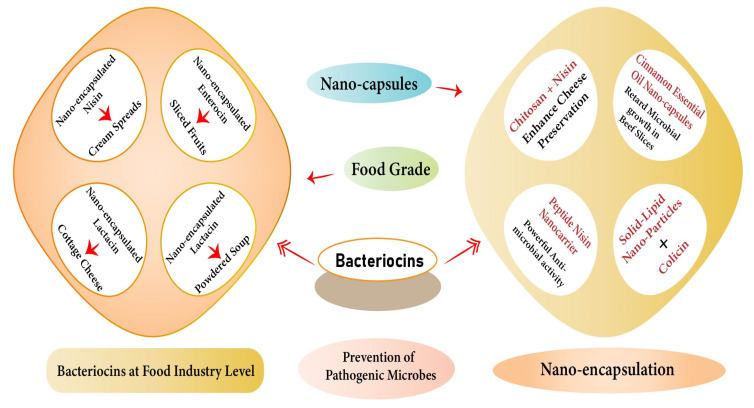
Graphical Abstract of Nano-encapsulated Bacteriocins in Food Safety.

Synthetic products based on chemicals are typically used to avoid spoilage and enhance the shelf life of foods. However, these chemicals have numerous adverse impacts on the health of humans. Compared with synthetic chemicals, naturally derived compounds are preferable and are applied by biopreservation [1,2]. Bacteriocins are antimicrobial peptides, therefore ribosomes are used to synthesize them. The main criterion for the classification of bacteriocins is their origin. Depending on whether they are produced by Gram-positive or Gram-negative bacteria, classes and subclasses of bacteriocins can be distinguished as shown in Figure 1. Lactic acid bacteria are utilized to produce bacteriocins that have the potential to retard the growth of pathogenic microorganisms [3]. Bacteriocins produced by lactic acid bacteria have been recognized as efficient at helping to maintain food safety and are generally recognized as safe (GRAS) [4,5]. Bacteriocins of lactic acid bacteria and their phylogenetically associated strains have a much broader antimicrobial activity spectrum [6].

Lactic acid bacteriocins are utilized in both the medical field and the food sector. Concerning food applications, they have been successfully applied as co-cultures or starters in experiments in the pilot study [7]. In the last decade, significant research has shown the effectiveness of bacteriocins in several branches of the food industry [8]. For example, one particular bacteriocin, nisin, is successfully used to improve the quality and storage period of milk and milk products [9,10]. Another study demonstrated that lactic acid bacteria and paracin with broad antimicrobial spectrum were successfully used to protect apple juice before bacterial contamination [11]. Enterocins are the broad-spectrum cyclic peptides against Gram-positive and Gram-negative bacteria, can be applied in milk products as well. Other products such as beef or fresh fish can also be protected with bacteriocins [12,13,14].

Metabolic products, bactericidal proteins, and antibiotic substances are produced by lactic acid bacteria. Lactic acid bacteria have the potential to inhibit numerous microorganisms in the food environment and exhibit vital antimicrobial characteristics related to food safety and preservation. In addition, strains of lactic acid bacteria provide health-enhancing capabilities regarding their potential in the medical sector. For instance, gastrointestinal pathogenic bacteria such as *Escherichia coli*, *Salmonella*, and *Helicobacter pylori* are mitigated by bacteriocins [15].

The functionality of bacteriocins produced by lactic acid bacteria is determined by many factors such as storage conditions, food matrix interaction, processing, pressure, temperature, and enzyme availability [16]. Effective nanoparticles are applied through nanoencapsulation to safeguard bacteriocins from degradation and deterioration. Nanoencapsulation is applied to bio-preservative bacteriocins to prevent enzyme degradation and to improve food product shelf life [17]. Nanoconjugates such as pediocin and nisin act as bacteriocins, and their applications provide unique packaging in food systems [18].

Gram-negative bacteriocins are used as proteins that efficiently play multi functions behavior to target pathogenic bacteria and bacterial species. Gram-positive bacteriocins are associated with advantageous properties such as the ability to deteriorate internal membranes and inhibit the growth of pathogenic microbes. Bacteriocins, either produced by Gram-negative or positive bacteria, always have the target of inhibiting the growth of other microorganisms in competition with the same environment. Hundreds of bacteriocins species have been discovered. The classification of bacteriocins is based on the sequence of amino acids, identified action mechanisms, and structure [19]. Starter cultures are used to produce bacteriocins, and exhibit potential applications in the dairy industry to protect fermented foods from the transmission of food pathogens [20]. Gram-positive bacteriocins are nano-encapsulated for application as bio-preservatives. Bacteriocin AS48 actively prevents pathogenic microbes such as *L. monocytogenes*, *S. aureus*, and *B. cereus* when used to prepare gelatin puddings, soy desserts, and baking cream. *L. monocytogenes* strains exist extensively in nature and are present in ready-to-eat meat. It has been recognized that Gram-positive bacteriocins inhibit or reduce the growth of *L. monocytogenes* during the processing of meat products [21].

Nanoencapsulation prevents proteolytic enzyme degradation and unwanted interactions with food components by enhancing food stability. Recently, several studies have revealed that bacteriocins encapsulation by nanoparticles improved the activity of peptides against multidrug-resistant bacteria and food spoilage microorganisms [22]. Anti-biofilms are manufactured by nanoencapsulation and have been regarded as a substantial mode for antimicrobial activity. Natural and synthetic nanoparticles are used in combination with bacteriocins to show improved efficacy in retarding the formation of biofilm and to assist in reducing antibiotic resistance [23]. Nanoparticles thus provide a wide range of characteristics to antimicrobial peptides such as bacteriocins with better functionality. These characteristics are physiological solution stability, a broad antibacterial spectrum, non-toxic nature, and ease of synthesis with less production cost and concentration [23].

Liposomes, silver nanoparticles, niosomes, nanovesicles, chitosan, solid lipid nanoparticles, phosphatidylcholine liposomes, and nanoliposomes are combined with Gram-negative and Gram-positive bacteriocins to develop nano-encapsulated bacteriocins for food systems. Nanoencapsulation promotes the antimicrobial action of bacteriocins to kill harmful bacteria, inhibits the interaction of pathogens directly with food substances, and ultimately extends the shelf life of food [24]. Nanoencapsulation is performed by several methods including nano-emulsification, nanoliposomes, electrospray, and formation of nanostructure with nanocrystals, nanostructure lipid carriers, and solid lipid nanoparticles [25].

This review addresses the importance of nanoencapsulation and highlights the bacteriocins of Gram-negative and Gram-positive bacteria and their potential application in various food industry systems. In addition, we review significant literature studies on the formulation of nano-encapsulated bacteriocins and the action mechanism of nano-encapsulated bacteriocins produced by lactic acid bacteria.

## 2. General Action Mechanism of Bacteriocins

Bacteriocins have been categorized into diverse classes, including Class I: lantibiotics that are stable in heat; Class II: non-lantibiotics (unmodified postranslationally); Subclass IIa: anti-Listeria-like and pediocin bacteriocins, Subclass IIb: two-peptide bacteriocins classified further into Subclass IIc: sec-dependent bacteriocins; Class III: non-lantibiotics that are labile to heat [26]. More bacteriocins have been separated and classified from lactic acid bacteria. Numerous bacteriocins have developed a position as effective antimicrobial agents due to their efficiency as food preservatives and provision of antagonistic impact to retard significant pathogens. The notable ones are nisin, pediocin, bulgarican, diplococcin, lactacins, acidophilin, plantaricins and helveticins [27].

The action mechanism of bacteriocins is focused on two diverse activity aspects, the physical interaction kinetics between susceptible cells and bacteriocin and certain biochemical lesions detection in target cells [28]. A wide variety of chemical structures permits bacteriocins to exhibit their impact on several important living cell functions (translation, transcription, biosynthesis of the cell wall, and replication, although most action is through the forming pores or membrane channels that disturb the potential energy [29]. It has been extensively postulated that the bacteriocin interacts with the target cell in two ways. The first way, which is perhaps reversible, resembles the bacteriocin’s physical adsorption using receptors in the cell. At this stage, bacteriocins are emerged to remove the cell intact; meanwhile, no perpetual physiological damage could be done. The second way causes irreversible pathological alternations because of certain biochemical cell damage [30].

In nature, microorganisms contribute to various mechanisms for establishing protection and interaction. These mechanisms are linked with the bacteriocins peptides production with prolonged antimicrobial activity. Gram-negative bacteriocin protein establishes an effective approach to prevent multi-drug-resistant bacteria through the folding of proteins into bacteria and by the production of certain species of antibiotics [31]. Protein bacteriocins capture pathways of nutrient uptake to cause cell death and translocation of the cell envelope. Their importation is strengthened by the parasitizing of intermembrane complexes of protein joined to the motive proton force, which causes the delivery of a toxic domain within the cell. A plethora of biophysical, genetic, biochemical, and structural procedures have taken place to uncover the components of the cell envelope involved in the import of bacteriocin [32].

Colicins are antibacterial proteins that are produced by some intestinal strains of bacteria, primarily situated in chromosomally encoded plasmids. These huge proteins contain three domains such as the amino-terminal domain that facilitates the transport of the target cell’s outer membrane, a receptor-binding domain that facilitates the transport within the periplasm, and a cytotoxic carboxy-terminal domain that shows the inhibitory impacts [33]. Three major action mechanisms for colicins have been described. These three mechanisms are the nuclease activity (e.g., target cell RNA/DNA), the formation of the pores that damage the integrity of the membrane, and murein synthesis inhibition [34]. *E. coli* is mainly responsible for the production of colicins which are bactericidal proteins that protect a colicinogenic plasmid. An immunity protein (imX or cxi), lysis proteins encoded gene and structural gene (cxa) are present in colicinogenic plasmids [32].

Colicin production regulation is facilitated by the response of SOS, which emerges to play a significant role in the host bacterial cell response to DNA damage. Three different action modes are provided by colicins: (1) the channels formation depends on voltage in the Gram-negative bacterial inner membrane, (2) the action of nuclease into the cytoplasm cells and (3) peptidoglycan degradation [32]. Genetic machinery available on chromosomes or plasmids is used to encode microcins. Microcins are categorized on the basis of three criteria: (1) the nature, localization, and presence of posttranslational modifications; (2) the organization of gene clusters; and (3) leader peptide sequences [35].

**Figure 2:**
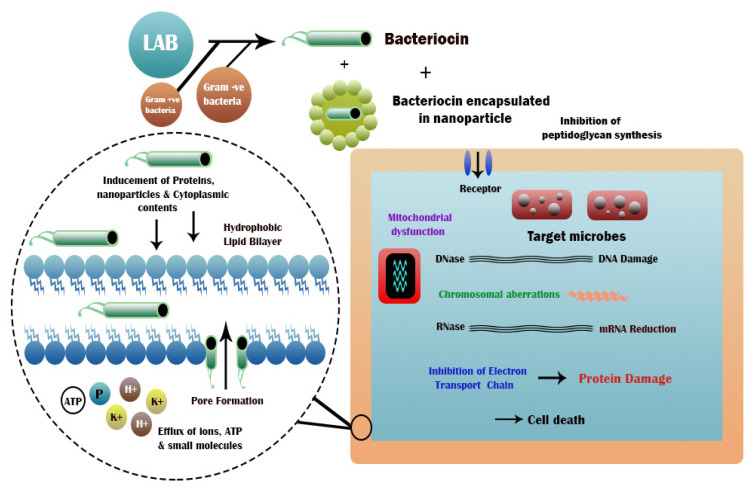
General method of bacteriocins synthesis, anti-bacterial activity, and action mechanism of bacteriocins [36,37].

Bacteriocins display action by joining with the conforming receptor on the sensitive bacteria surface to retard bacterial growth. Bactericidal action mechanisms involve the peptidoglycanase type, RNase, and DNase function of the nuclease type and pore-developing type. Subtilisin A has an unmodified bacteriocin peptide structure and a low molecular weight. Colicin Ia and bacteriocin AS-48 have modified bacteriocin protein and peptide structures and low molecular weight as well. The strain is grown on the soft agar of sensitive bacteria LB to produce bacteriocin, and production zone occurs around the strains that produce bacteriocin. However, strains that do not produce bacteriocin have no inhibition zone [36]. The general bacteriocin mechanisms involve bacterial growth reduction comprising bacteriocin binding, bacteriocin translocation, enzyme activity modulation, and pore formation of the cytoplasmic membrane [37]. The general method of bacteriocins synthesis, anti-bacterial activity, and action mechanism of bacteriocins is shown in Figure 2.

## 3. Formulation of Nano-Encapsulated Bacteriocins

Nanoencapsulation is well defined as a novel technology to package constituents in minute assembly, with the usage of methods such as nano emulsification, nano structuration, and nanocomposite. It carries the final functionality of the product (containing limited core release) to maintain product quality expectations during storage [38]. Modification of nanomaterials can occur in the form of nanospheres, nanorods, nanoparticles, and nano frames and play their role in applications specifically with the means of biomedicine, electronic, solar energy conversion, environmental applications, water treatment, and some catalysis processes [39]. The principle of encapsulation includes core material or solid matrix usage to isolate bacterial cells, bioactive components, and other concerned agents from the environment. Nanoencapsulates are usually semi-permeable and spherical networks ranging in size from 10^−9^ m to 10^−6^ m [40,41].

### 3.1. Chitosan-Encapsulated Nisin

Nisin acts as a bacteriocin, also known as a food additive (E234) obtained from *Lactococcus lactis*, and exhibits antimicrobial properties and potential applications in food. Nisin imparts strong sporostatic and bactericidal actions to retard Gram-positive bacterial growth. Perhaps the bactericidal activity shown by nisin to inhibit Gram-negative bacterial growth is restricted due to nisin’s inaccessibility to the plasma membrane [42]. A liposome is applied as a shell material by encapsulation but faces numerous disadvantages, comprising increased oxidative degradation susceptibility, increased phospholipids cost, and joined liposomes sedimentation during storage [43]. Nonionic surfactant niosomes or vesicles are better substances for shell material by encapsulation in the food area due to their low surfactant material cost compared to the more expensive liposome materials. Numerous nonionic surfactant classes such as glucosyl dialkyl ethers, polyglycerol alkyl ethers, polyoxyethylene alkyl esters, and ether and crown ether are added in niosomes preparation [44].

Nano-encapsulated nisin imparts significant actions to suppress the growth of *S. aureus* in raw milk for 24 h and pasteurized samples for 48 h as compared to free nisin, whose action cannot exceed the storage life of milk and remains 14 and 24 h, respectively [45]. Chitosan is a biodegradable, nontoxic copolymer that contains units of N-acetyle-D-glucosamine and D-glucosamine from the deacetylation of chitin in hot alkali availability. Chitosan is a nontoxic, biocompatible polymer that has the cohesive capability to act as an antimicrobial peptide and is developed as a nanoparticle-based vehicle. Chitosan also shows antimicrobial activity to retard numerous pathogenic and spoilage microorganisms from Gram-negative and Gram-positive bacteria, yeasts, and molds. The antimicrobial effect shown by chitosan is based on microorganism type, pH value, deacetylation degree, and molecular weight. Chitosan-encapsulated nisin is utilized to retard microbial growth and thus increase the shelf-life of food products [46,47,48].

### 3.2. Liposome-Encapsulated Pediocin

Nano-delivery systems containing carbohydrates, lipids, protein surfactants, and polymers have been modified to enhance and stabilize the biological activity of bacteriocins. For example, liposomes are comprised of spherical structures with phospholipids encompassing an aqueous medium through single or multiple bilayer membranes, and their size ranges from 10^−9^ to 10^−6^ nm. Liposomes are biodegradable and nontoxic agents appropriate for encapsulating both hydrophobic and hydrophilic substances [49]. The encapsulation of bacteriocins has been a significant development in utilizing natural antimicrobials in the area of food science. The activity of bacteriocins is influenced by various factors such as solubility changes, bacteriocins’ charge, inactivation through proteases, and bacteriocins binding to food components. A liposome is potentially used to encapsulate pediocin to sustain its antimicrobial activity for a long duration. Silver nanoparticles have antimicrobial efficiency, which can be achieved and increased by encapsulation with antimicrobial agents, namely bacteriocins. Nanotechnology is a novel, fabricated, and a new platform to develop nano-structured substances possessing antimicrobial activities [50].

*Pediococcus acidilactici ITV26* is potentially utilized to produce pediocin bacteriocin which is a bioconservative and exhibits antilisterial activity. Inactivation of pediocin may occur when available in foods in free form. The binding/interaction of proteolytic enzymes with a few food substances is an antimicrobial activity-affected factor. Therefore, encapsulation must be applied to protect these peptides and limit their release into liposomes. Researchers must express interest in Class II of bacteriocin, namely pediocin, because of its thermostability and strong action to prevent *Listeria monocytogenes* [51]. Liposomes contain more than one phospholipid bilayer and are known as spherical vesicles. Liposomes also possess active peptides and are recognized as bacteriocins by enclosing peptides between phospholipids bilayers and into the aqueous center due to their amphiphilic nature. Liposomal vesicles contain molecules of amphiphilic nature, and their structure can be described as molecules with a non-polar tail, a polar head, a concentric series, and two hydrophobic chains that are found on each molecule [52]. Class IIa bacteriocin contains pediocin in the gelatinous form to reduce *Listeria* infection from the consumption of hot dogs. Additionally, phosphatidylcholine nanovesicles are loaded with pediocin AcH possessing high antimicrobial activity, high efficiency for entrapment (80%), and high stability [53].

Nanoliposomes have diverse compositions and structures and are regarded as versatile tools for encapsulation technologies. Liposomes consist of spherical, closed structures and are made up of curved lipid bilayers. These lipid bilayers enclose the surrounding solvent in their interior part. Major liposomal substances are phospholipids containing amphiphilic molecules with a water-soluble section consisting of a hydrophilic head and a lipid-soluble section consisting of a hydrophobic tail which forms the composition of liposomes [54]. This phospholipid property provides distinctive benefits to liposomes, such as enclosing themselves in aqueous media and establishing a perfect carrier system. Liposomes provide applications in cosmetics, pharmaceuticals, and most importantly, food. Liposomes are extensively utilized as carrier systems based on lipids in the food area, predominantly antimicrobial formulations by liposomes. Phosphatidylcholine, lecithin, nanoliposomes; guar gum, and alginate capsules; plus guar gum and alginate hybrid capsules are combined with nanoliposomes to form nano liposomal encapsulated pediocin [55].

### 3.3. Solid Lipid Nano-Encapsulated Colicin

Colicins are sensitive to heat and protease enzymes with more molecular weight (30–80 kDa). They are known as bactericidal proteins produced by the strains of *E. coli* and have one colicinogenic plasmid. Colicins compounds are primarily used and studied as model systems to study the bacteriocin evolution functions and structures [56]. Synthesis of colicin is lethal for the production of cells because of lysis protein co-expression. Colicins are categorized into three major groups: degrading peptidoglycan, pore-forming, and nuclease based on the interaction mechanism with the target cell. The accomplishment of uptake of colicin due to the target cell is conducted by involved receptors in nutrients transport such as Fiu-bound iron, Tsx receptor nucleosides, cobalamin receptor BtuB vitamin B12, siderophore FepA-, FhuA-, and Cir- [57]. Colicins, lantibiocins, and enterocins are broad-spectrum bacteriocins that affect the larger bacterial genera group. Moreover, porin proteins are used by colicins to limit the passive diffusion of amino acids, phosphates, and sugars with the aid of the outer membrane [58].

Gram-negative bacteria have the potential to manufacture an extensive bacteriocins variety that is named specifically after the Klebsiella pneumoniae klebicins genus or species-producing bacteriocins such as *Serratia marcescens*-producing marcescins, *E. coli*-producing colicins, *Hafnia alvei*-producing alveicins, and *Enterobacter cloacae*-producing cloacins. Pseudomonads are generally associated with the production of pyocins [59]. Gram-negative bacteria mainly produce bacteriocins from Enterobacteriaceae. They are categorized into two major families such as colicins having more molecular mass (30–80 kDa) and microcins having less molecular mass (between 1 and 10 kDa) peptides [60]. SOS response regulon causes the mitigation in colicins production and emerges to play a key role in bacteria to DNA damage response. Microcins are hydrophobic peptides with increased stability and maximum stress production, mainly depletion of nutrients. Microcins and colicins are significantly available in *Escherichia coli*. However, many species of Gram-negative bacteria produce bacteriocin-like constituents [32]. Major bacteriocins of Gram-ve bacteria are colicins [33], pyocins [61] and microcins [62].

Colicin is a representative Gram-negative bacteriocin mainly produced by *Escherichia coli*. It is a protein with maximum molecular weight and it is used to reduce several Gram-negative bacteria. Mice-treated streptomycin is survived by *E. coli* rather than the non-colicin. The competitive benefit is gained, which permits the existence of the strain produced by bacteriocin. Multi-strain and even in multispecies, probiotics are superior and endorsed to produce bacteriocin [63,64]. Solid lipid nanoparticles (SLN) compose a suitable solid triglyceride core for the slow formulation of drug release. Colicins and nisin can be protected by SLN against degradation, increasing the anti-bacterial activity for the duration. Rather than free colicin and nisin, SLN enclosing colicin and nisin exhibited a great ability to retard *L. plantarum TISTR 850* for up to 15 days and *L. monocytogenes DMST 2871* for 20 days [65].

### 3.4. Nano-Encapsulated Microcins

The third type of bacteriocins produced by Gram-negative bacteria is microcins. Microcin is a bacteriocin that is synthesized by *E. coli* and has similarities with Gram-positive bacteriocins concerning thermal stability, protease resistance, and pH [66]. Microcins display dominant anti-bacterial activity and depend on subtle penetration mechanisms by the Gram-negative inner and outer bacterial membranes. Siderophore-microcins are involved in the binding of receptors to avoid the outer membrane in the transportation of iron. Cyclic microcin J25 is formed by the availability of an N-terminal macrolactam ring and utilizes the receptor of hydroxamate and intracellular protein SbmA membrane. Microcin C is synthesized as heptapeptide adenylate, requires external porins membrane and transporters of ABC membrane, and transforms into an adenylate that is a non-hydrolyzable aspartyl equivalent in the cytoplasm [67].

Microcin N (McnN) acts as antimicrobial peptides which have been examined for their capability to combat these foodstuff pathogens and selected for aquatic and human consumption. Bacteriocin McnN is synthesized by a non-pathogenic *E. coli* strain that shows activity to combat *Salmonella and E. coli* species [68]. Microcins from Class I contain Mirocin C7–C51, Microcin J25, and Microcin B. Microcin peptides from Class IIa are encoded by a plasmid including linkages of disulfide and require no modification of post-translation. Microcin peptides from Class IIb are chromosomally encoded peptides and undergo a general post-translation siderophore by C terminal modification [69]. Currently, antimicrobial peptides (AMPs) are known as host defense peptides (HDPs) and are interesting due to their potential for substitution in the novel strategies for resistance offered by bacteria to combat antibiotics in diseases and infections. However, several drawbacks are linked to antimicrobial peptides due to their lesser bioavailability, lesser solubility, and easy protease degradability that controls their antimicrobial usage. Vehicles used for AMPs delivery include polymers, micelles, nanoparticles, dendrimers, carbon nanotubes, and other system types which permit the AMPs to be used as a substitute for antibiotic treatment [70].

### 3.5. Nano-Encapsulated Lantibiotics

Gram-positive bacteria are categorized into four broad groups: lantibiotics, large proteins, non-modified small peptides, and cyclic peptides [71]. Gram-positive bacteriocins are classified into two major classes: lantibiotics are included in Class I while unmodified postranslationally small bacteriocins are included in Class II. Lantibiotics are known as peptides that include wide modifications of post-translational and possess methyllanthionine and/or lanthionine residues. In the previous literature, it has been elucidated that this ribosomally and postranslationally synthesized class of modified peptides (RiPPs) is considerably unusual [19].

Gram-positive bacteriocins establish an extensive anti-bacterial spectrum as compared to other bacteriocins. A peptidoglycan multilayered thick wall is generally attributed to providing a large spectrum rather than an outer membrane. Small peptide penetration is enabled by outer organization regardless of any binding of the receptor [72]. The third bacteriocins class formerly comprised bacteriolysins, currently recognized tailocins, and large anti-bacterial proteins of Gram-positive bacteria (up to 10 kDa). Lantibiotics include typical amino acids such as methylanthionine (MeLan), dehydrobutyrin (Dhb), D-alanine (D-Ala), lanthionine (Lan), and dehydroalanine (Dha) [73].

Lantibiotics are peptides that have a reduced molecular weight of approximately 5 kDa and include residues of methyllanthionine and/or lanthionine. Lantibiotics provide stability to the bacteriocin structure and resistance to protease action. Lantipeptides are classified into four classes depending on the biosynthesis specifics, in which two compounds possess anti-bacterial activity. Lantibiotics are categorized into three types depending on the structure features, such as AI, AII, and B equivalent linear bacteriocins, combined bacteriocins, and globular bacteriocins conformation [74].

AI-type lantibiotics contain microbisporicin, nisin, and epilancin 15×. Their anti-bacterial action depends on the reduction of cell wall production due to the N-terminal domain bacteriocin binding to lipid II, also known as peptidoglycan precursor. Moreover, the domain C-terminal plays a significant role in pores formation that causes potential membrane violation. Type-B lantibiotics include mersacidin, cinnamicin, and actagardin with a globular tertiary compact structure [75]. An isolated group contains two lantibiotic components that provide synergistic anti-bacterial action. The most-reported two components of lantibiotic are lactacin 3147 which includes type A1 β-peptide (LtnA2) and type B α-peptide (LtnA1). Lactacin 3147 shows anti-bacterial action and is examined in the targeted cell membrane by the formation of the pores. Lantibiotics exhibit strong activity and structural diversity in combatting Gram-positive pathogens. For instance, nisin has been utilized as an effective food preservative for the last 50 years. Numerous novel lantibiotics are presently undergoing clinical trials to examine their antimicrobial potential [76].

### 3.6. Nano-Encapsulated Peptides

The most interesting and stable form of bacteriocins are cyclic structure bacteriocins for practical applications. The compounds of this class are glycocins, lasso peptides, and peptide bonds with “head-to-tail” bacteriocins. Lasso peptides are named thus due to tertiary structure characteristics which are observed by the isopeptide bond formation between the N-terminal macrolactam amine ring and the aspartic acids or glutamic carboxylic acid residue at the 7, 8, or 9 positions of the C-terminal tail peptide sequence. Recently, the three lasso peptide bacteriocin structures have been synthesized by Gram-positive bacteria that have been categorized as follows: streptomonomycin from Streptomonospora alba, svicenin from Streptomyces sviceus, and lariatin A from Rhodococcus jostii [77].

The highly stable structures of lasso peptides are resistant to high temperatures and enzyme action. Properties of lasso peptides are lost during linearization; thus, it is not required to synthesize active bacteriocins chemically. At the receptors, every lasso peptide is target-specific according to the bacterial specie. As a result, streptomonomycin is a prevailing bacillus species inhibitor. It is supposed that the activity of the bacteria resulted in the interaction of the WalR protein for division and metabolism in the cell wall. Consequently, regardless of the other classes of bacteriocins that have comparable action mechanisms due to structural features, three-dimension structure of every peptide produces a definite signaling molecule [78]. Bioactive peptides by nanoencapsulation thus enhance bioavailability and defend stability during distribution, processing, and storage. The result is that consumers are presented with food with potential health benefits, and the stability of these peptides is also improved [79,80]. Studies on the formulation of nano-encapsulated bacteriocins through different nanoparticles are shown in Table 1.

**Table 1:** Reported Studies on the Formulation of Nano-encapsulated Bacteriocins.

LAB Bacteriocins	Purification of Bacteriocins	Nanoparticles	Applied Encapsulation Technique	Nanoencapsulation Conditions	Effective against Bacteria	Effect of Nanoformulation and Applied Technique	References
Nisin	Milk fat globule membrane (MFGM) phospholipids-based nanostructures	Rhamnolipids (RLs)	Ultrasonication-assisted self-assembly method	Sonicated for 30 min	Prevent *Escherichia coli* and *Listeria monocytogenes*	Enhanced cheese preservation to prevent the foodborne pathogens	[81]
Nisin	------------	Cinnamon essential oil nanocapsules (CEO-NPs)	----------	------------	Retard the microbial growth and decreased lipid oxidation	Increased storage of beef slices for 15 days	[82]
Peptide nisin	Dissolution of 3 mg/mL nisin in acetic acid solution at pH 4.0 to obtain a stock solution of nisin	Nanocarrier based on polysaccharide with curcumin	Ultrafiltration tubes, Magnetic stirring	Stirring for 30 min at 25 °C with final 4.0 pH	14.00 mm on *B. subtilis* and 12.97 mm on *L. monocytogenes*	Nanocarriers were fabricated to provide multifunctional potential in the food and show powerful antimicrobial activity	[83]
Nisin	1 mg/mL nisin form Film-forming solutions (FFS) at 600 rpm by stirring at room temperature for 40 min	Nano-rhamnosomes	Field Emission Scanning Electron Microscope (FE-SEM)	10 kV accelerated voltage	Inhibit the *E. coli* and *L. monocytogenes* growth	Prolonged bioactive preservation of food by broad-spectrum antimicrobial activity to combat Gram-negative and Gram-positive foodborne pathogens	[84]
Nisin	Digestion of protein with trypsin 20 μg and incubation at 37 °C, dried for 16 h, and storage temp is −20 °C	Phosphatidylcholine liposomes	Sonication and Thin-film hydration method	Dried through thin film for 24 h in a desiccator and nisin solution in phosphate buffer (10 mm) addition at 100 μg/mL and sonicated for 3 min at 55 kHz	Liposome-encapsulated nisin decreases stresses and lowers the occurrence of *L. monocytogenes*	Liposome encapsulation might be an effective approach to prevent nisin resistance	[85]
Nisin	Nisin was dissolved in the water phase with soy oil (20 mL) and gelatin (1%)	Polyacrylate Sodium (PAAS) and polyvinyl alcohol (PVA)	Sonication, Response Surface Methodology, Electrospinning	Ultra-sonication time (15 min) and 15%, centrifugation (6000 rpm) at 4 °C for 5 min after freeze-drying for 48 h, at −50 °C	*Staphylococcus aureus* and *Escherichia coli* growth was prevented for 16 days	Nanofiber can potentially retard food microorganisms’ activity in food and prolong the strawberry’s shelf life	[86]
Plantaricin	Sodium sulfate method	Silver nanoparticles	------------	------------	Showed inhibitory activity towards *Listeria monocytogenes*	The stability period got increased from 5 days to 60 days	[87]
Bacteriocin	-----------	Au-zein-based nanomats	Electrospinning method	Samples were stored at (4 ± 1 °C)	~1 log CFU/g reduction of bacteria	Reduced the growth of mesophilic aerobic bacteria in skinless fish fillets	[88]
Nisin	----------	Nanofibers (NP) with polyethylene (PE) packs	Electrospinning method	1.2 mL/h, 8 cm, and 20 kV	Total mesophilic bacteria from 5.03 to 3.52 log CFU/g, Lactic acid bacteria from 3.22 to 2.02 log CFU/g	Prevention of off-odor and reduction of microbial growth in rainbow trout fillets	[89]

## 4. Advantage of Antimicrobial Peptides at the Food Industry Scale

The potential application of nano-encapsulated antimicrobial peptides such as bacteriocins at the food industrial level needs to be encouraged. Several studies have demonstrated the possible use of lactic acid bacteria in the manufacturing of cheese. In this process, the active conversion of lactose to lactic acid produces bacteriocins to change the composition of complex cheese microflora and inhibition of pathogenic bacteria or adventitious spoilage. Particularly, the Joint Food and Agriculture Organization/World Health Organization Expert Committee on Food Additives in 1969 claimed that nisin is regarded as safe to be potentially used in food. In 1983, number E234 was allocated to this bacteriocin; it was added to the European food additive list and, finally, US Food and Drug Agency approved it to be used effectively in processed, pasteurized cheese spreads in 1988. Bacteriocins that are produced from lactic acid bacteria are generally utilized to preserve food and inhibit the spoilage and pathogenic microbes in food products and provide significant antimicrobial dimensions [90].

Enterocin AS-48 treatment deactivates *L. monocytogenes* cells inoculated on the slices and surface fruit and completely or partially disables *S. Aureus* in several sauces of fruit and vegetable [91]. A wild strain of *Streptococcus thermophilus ACA-DC 0001* was isolated from traditional products such as Greek yogurt used to produce Thermophilin ST-1 [92]. Thermophilin ST-1 has an inhibitory impact on numerous foodborne pathogens, food spoilage microorganisms, lactic acid bacteria, and on a few phytopathogens of Gram-negative bacteria including the following: *Enterococcus faecalis EF1*, *Xanthomonas campestris BPIC 1660*, *Listeria innocua BL 86/20*, *Erwinia rubrifasciens BPIC 1710*, *Pseudomonas syringae BPIC 1549* and *Staphylococcus aureus ATCC 29996*. Antimicrobial substances are sensitive to increased alkaline and acidic conditions, to proteolytic enzymes, specifically trypsin and pronase, heat-labile at 60 °C for 10 min, and exhibit a bactericidal action mode to prevent the *Lactococcus lactis ssp. cremoris CNRZ-117* indicator strain [92].

Lactacin has an active antimicrobial potential that can be utilized in food products such as powdered soup, baby milk, cottage cheese, and yogurt [57]. It has been reported that lactacin is non-immunogenic and nontoxic and mitigates the occurrence of infection. Lactacin is produced by lactic acid bacteria (*Lactococcus lactis 3147*), which is utilized significantly to prevent the development of several types of Gram-positive microorganisms such as *Pediococcus acidilactici*, *Clostridium botulinum*, *Listeria monocytogenes*, *Clostridium sporogenes*, *Staphylococcus aureus*, *Enterococcus faecalis*, and *Bacillus* spp. [93,94]. 

Nanoencapsulation of bacteriocins is the successful development through regulatory approval from initial biological experimentation and observation to commercial application. In recent years, it has stimulated a new approach in bacteriocins research for the industrial model and market innovations. It is a fact that without understanding the nature and mode of action, bacteriocins can be problematic in food. To reduce these disadvantages, nanoencapsulation can be an effective approach in various important commercial and imaginative applications. The core matrix and structurally enhanced nanocapsule probably have the potential in food applications, especially to encapsulate the antimicrobial peptides that are produced by food-grade LAB. Most importantly, it is more likely to meet regulatory approval specifically to their origin for their introduction into fermented foods without any purification or concentration [4].

## 5. Conclusions

This review highlighted the integral role, features, action mechanism and nanoencapsulation method of bacteriocins produced by lactic acid bacteria. Nowadays, food spoilage is a major concern in the food industry. The trend of applying natural and chemical-free preservatives has been increasing due to consumer concerns regarding the side effects of artificial preservatives as well as due to their chronic health effects. Bacteriocins are more efficient, anti-bacterial agents with fewer adverse effects. However, challenges and limitations are associated with bacteriocins usage as anti-bacterial agents or bio-preservatives in the food industry. Currently, available bacteriocin peptides have proven their efficacy, and various examples have been provided to ensure their commercial applications in the food sector. For example, nanoencapsulation acts as a suitable strategy to preserve biological activities and enhance the stability of nanoparticles when successfully applied to a food product. The efficient technique of nanoencapsulation uses nanomaterials that enhance the antimicrobial potential of bacteriocin. Moreover, these nanomaterials interact with bacteriocins to develop nano-formulated bacteriocins as well as establish the mechanism of action against targeted microorganisms which have been explained.

## 6. Future Recommendations

Although nano-encapsulated bacteriocins have been applied in the food industry efficiently, additional research effort is needed to encapsulate nutraceuticals and to explore them as nutraceutical carriers [49]. It is of great importance to conduct more studies on nano-encapsulated enterocin, lactacin, thermophilin, lacidin, sakacin, and bulgaricin [6]. Novel research approach is required to produce bacteriocins from genetically modified organisms and to develop suitable conditions for their application through nanoencapsulation methods [95]. Further studies are also needed to evaluate the in vivo efficiency and safety of peptides. Identifying the gene of new recombinant bacteriocins has become important by using polymerase chain reaction techniques to determine their applicability in food and medicine [36]. The bioavailability and stability of bacteriocins throughout the food chain during production, processing, distribution and storage is significant through nanoencapsulation method to modify the food with health benefits.
